# Raciborski’s Elements of Diagnosis

**Published:** 1841-05

**Authors:** 


RACIBORSKI'S ELEMENTS OF DIAGNOSIS.

We are happy to learn that a translation of this excellent work, decidedly the best on the subject in any language, will shortly appear from the pen of Mr. Hagan, a student of the Louisville Medical Institute. It will be comprised in an octavo volume of about 550 pages, and be published at an early period. From his intimate knowledge of the French language, and familiar acquaintance with the subject of the work, there is every reason to believe that Mr. Hagan will furnish a most excellent translation. S. D. G.

